# Protective Response in Experimental Paracoccidioidomycosis Elicited by Extracellular Vesicles Containing Antigens of *Paracoccidioides brasiliensis*

**DOI:** 10.3390/cells10071813

**Published:** 2021-07-17

**Authors:** Ludmila Matos Baltazar, Gabriela Fior Ribeiro, Gustavo J. Freitas, Celso Martins Queiroz-Junior, Caio Tavares Fagundes, Carlos Chaves-Olórtegui, Mauro Martins Teixeira, Daniele G. Souza

**Affiliations:** 1 Laboratório de Interação Microrganismo Hospedeiro, Departamento de Microbiologia, Instituto de Ciências Biológicas, Universidade Federal de Minas Gerais, 6627, Antonio Carlos Ave, Belo Horizonte, Minas Gerais 31270-901, Brazil; ludmilabaltazar@gmail.com (L.M.B.); gabrielafior@hotmail.com (G.F.R.); caio.fagundes@gmail.com (C.T.F.); 2 Departamento de Microbiologia, Instituto de Ciências Biológicas, Universidade Federal de Minas Gerais, 6627, Antonio Carlos Ave, Belo Horizonte, Minas Gerais 31270-901, Brazil; gustavofreitascota@ufmg.br; 3 Departamento de Morfologia, Instituto de Ciências Biológicas, Universidade Federal de Minas Gerais, 6627, Antonio Carlos Ave, Belo Horizonte, Minas Gerais 31270-901, Brazil; cmqj@ufmg.br; 4 Departamento de Bioquímica e Imunologia, Instituto de Ciências Biológicas, Universidade Federal de Minas Gerais, 6627, Antonio Carlos Ave, Belo Horizonte, Minas Gerais 31270-901, Brazil; olortegi@icb.ufmg.br (C.C.-O.); mmtex@ufmg.br (M.M.T.)

**Keywords:** extracellular vesicles, *Paracoccidioides brasiliensis*, immunization, host response

## Abstract

Paracoccidioidomycosis (PCM) is a systemic disease caused by *Paracoccidioides* spp. PCM is endemic in Latin America and most cases are registered in Brazil. This mycosis affects mainly the lungs, but can also spread to other tissues and organs, including the liver. Several approaches have been investigated to improve treatment effectiveness and protection against the disease. Extracellular vesicles (EVs) are good antigen delivery vehicles. The present work aims to investigate the use of EVs derived from *Paracoccidioides brasiliensis* as an immunization tool in a murine model of PCM. For this, male C57BL/6 were immunized with two doses of EVs plus adjuvant and then infected with *P. brasiliensis*. EV immunization induced IgM and IgG in vivo and cytokine production by splenocytes ex vivo. Further, immunization with EVs had a positive effect on mice infected with *P. brasiliensis*, as it induced activated T lymphocytes and NKT cell mobilization to the infected lungs, improved production of proinflammatory cytokines and the histopathological profile, and reduced fungal burden. Therefore, the present study shows a new role for *P. brasiliensis* EVs in the presence of adjuvant as modulators of the host immune system, suggesting their utility as immunizing agents.

## 1. Introduction

Paracoccioidomycosis (PCM) is a systemic mycosis caused by the dimorphic fungi *Paracoccidioides brasiliensis* and *Paracoccidioides lutzii*. This mycosis is endemic to Latin America, with the highest number of reported cases in Brazil, which has an annual incidence of 0.1–3.7 cases per 100,000 individuals [1]. Additionally, the disease affects primarily male rural workers due to the activities associated with their work requirements, as well as the pathogen’s route of infection. This indicates an important characteristic of PCM—the fact that it affects mainly vulnerable and low-income populations [1,2].

The infection occurs via mycelial fragments and/or conidia inhalation, which switch from the filamentous form (the transmissible form) to yeast form (the pathogenic form) in the host. Classically, the clinical forms of PCM are divided into acute/subacute (juvenile) and chronic (adult) manifestations, the latter representing 74% to 96% of cases [1]. PCM control depends on the cellular response of the host, with T cell participation considered particularly important, although dormant forms of the fungus may still survive within granulomas. In this regard, a Th1 response pattern and compact granuloma formation are described as pivotal to control fungal replication [1]. Patients that develop PCM, however, exhibit a deficient Th1 response [2,3]. Additionally, a Th2 and Th9 response without the formation of compact granulomas has been observed in individuals who present the severe form of this disease [2,3]. 

Several approaches have been investigated to improve treatment effectiveness and protection against the disease [4,5,6,7]. The first candidate for a vaccine against PCM was the peptide P10, a 15-amino acid peptide (QTLIAIHTLAIRYAN) derived from a 43 kDa glycoprotein (gp43) which is the immunodominant antigen of *P. brasiliensis* [4]. Since then, other strategies have been evaluated, such as a DNA vaccine (pcDNA3-P10), immunogenic virus-like particles (VLP) carrying multiple copies of the P10 peptide, and therapy with three doses of DNA vaccine pVAX1-PB_HSP60, built with the pVAX1 vector subcloned with the gene of PbHsp60 [5,6,7]. 

Extracellular vesicles (EVs) are bilayer membrane structures released by cells of both prokaryotic and eukaryotic organisms, such as fungi [8]. Studies of EVs released by fungal cells indicate their use to transport and deliver substances to the extracellular environment [9]. Moreover, it has been recently recognized that EVs may modulate host immune responses [9,10,11,12]. For instance, production of EVs by *P. brasiliensis* cells and their in vitro effects on macrophage modulation have been previously reported [13,14]. These studies showed that *P. brasiliensis*-derived EV promote M1 polarization in macrophages, enhancing its fungicidal activity and the production of proinflammatory mediators [14]. Therefore, we hypothesized that EVs from *P. brasiliensis* cells could induce the activation of the host immune system and generate a protective response against *P. brasiliensis* infection. 

In the present study, we investigated the effectiveness of EVs from *P. brasiliensis* on mouse immunization and its subsequent effects on fungal burden, production of inflammatory mediators, and activation of immune cells. Our results demonstrate that immunization with EVs from *P. brasiliensis* in the presence of adjuvant protects mice against PCM infection leading to reduced fungal burden, enhanced production of proinflammatory mediators and activated T lymphocytes, and Natural Killer T (NKT) cells mobilization, suggesting its potential use in the immunization against *P. brasiliensis* infection. 

## 2. Material and Methods

### 2.1. Fungal Strain

EVs were obtained from the *P. brasiliensis* isolate 18 [15,16]. Infection of the murine host was performed using the *P. brasiliensis* 18 recovered from male C57BL/6 mice. Both replications of the original isolate and the isolate which was recovered from mouse infections were maintained in the yeast phase at 37 °C in YPD media slants (1% yeast extract, 2% casein peptone, 2% glucose, 2% bacteriological agar, pH 6.5). 

### 2.2. Extracellular Vesicles Purification

EVs were purified according to the protocol described by Baltazar et al. [12], with minor modifications. For isolation of EVs, yeast cells were transferred from 7-day-old slants YPD medium into Erlenmeyer flasks containing 0.5 L of YPD broth (1% yeast extract, 2% casein peptone, 2% glucose, pH 6.5). The concentration of fungal cells was adjusted to 1 × 10^6^ cells/mL in a volume of 0.5 L and cultivated for 7 days at 37 °C with constant shaking at 120 rpm (log-phase). To maintain the log phase, 0.25 L of fresh media was added to the cells every 48 h, i.e., 0.25 L of media was added on days 3 and 5. This resulted in a final volume of 1 L at the end of the 7 days. Yeast cells were removed by centrifugation at 3000 rpm for 10 min at 4 °C. Then, yeast was filtered with a 0.45 µm-pore-size filter membrane (Sartorius, Goettingen, NI, German. Cell-free supernatant was concentrated using an Amicon ultrafiltration system with a 100-kDa cutoff membrane of filtration (Millipore, Burlington, MA, USA). After filtration, the remaining EVs were washed out from the membrane surface with phosphate-buffered saline (PBS). Next, the collected vesicles were ultracentrifuged at 100,000× *g* for 1 h at 4 °C. The supernatant was removed, and the pellets were suspended in 0.1 mL of filtered PBS and centrifuged in the same conditions. The pellet of EVs was resuspended in 0.1 mL of PBS containing protease inhibitor cocktail (Sigma-Aldrich, Darmstadt, HE, Germany). Protein quantification in the vesicles was performed using bicinchoninic acid (Sigma-Aldrich, Darmstadt, HE, Germany) according to manufacturer instructions. 

### 2.3. Mice and Ethics Statement 

All the protocols, including immunization and mouse infection, were performed using healthy immunocompetent male C57BL/6 mice (4 weeks old, 19–20 g) that had not undergone any previous procedure. Animals were purchased from Biotério Central, UFMG, Brazil and kept in the Biotério de Imunofarmacologia, housed in clean cages with access to food and water ad libidum and 12 h light/dark cycles. Six mice were assigned to each cage. Mice were individually marked on their tails, considering the total number of mice per experiment, and then randomly allocated in each experimental group. This procedure is important to minimize potential confounding factors such as the order of treatments and measurements, or animal/cage location. Mice in the same cage were part of the same treatment. Each experimental group was given a numbered code to allow for blinding and only one person was aware of the group allocations at different stages of the experiment. Mouse experiments were approved by the Ethics Commission on Animal Use (CEUA) from Federal University of Minas Gerais (UFMG), Brazil, (Protocol 298/2019).

### 2.4. Immunization Protocol

Experimental groups (six mice each) were subcutaneously immunized with either 100 ng or 1000 ng of EV protein per animal, with or without MONTANIDE^TM^ ISA50 V2 adjuvant (SEPPIC), in a final volume of 0.1 mL/mouse. The control group was injected with PBS. Immunized groups were injected subcutaneously at day 0, and a booster injection was administered fourteen-days after the first dose. Adjuvant was included only in the first dose, not the booster. Blood from the tail vein was collected for antibody production quantification at 28, 35, and 42 days post-immunization. Mice were euthanized on the 42nd day after the first immunization, their spleens were collected and splenocytes were isolated for subsequent use in cell proliferation assays followed by incubation with *P. brasiliensis* EV protein to assess immunological memory. 

### 2.5. Splenocyte Response Assay

Spleens were aseptically removed from immunized mice and red blood cells were disrupted by osmotic lysis. Splenocytes (2.0 × 10^5^ viable cells) were incubated in RPMI 1640 medium supplemented with 10% Fetal Bovine Serum (FBS) for 48 h at 37 °C and 5% CO_2_. Positive and negative controls were cells stimulated with Concanavalin A at a concentration of 2.0 µg/mL and non-stimulated cells, respectively. In addition, splenocytes from each immunized group were stimulated ex vivo with 100 ng of EV protein. After the incubation period, the supernatant was collected for quantification of the cytokine IFN-γ.

### 2.6. Mice Infection 

On day 42-post-immunization, mice were intratracheally infected with 1 × 10^6^ *P. brasiliensis* yeast cells/mouse after anesthesia with a solution containing ketamine hydrochloride (80 mg/kg) and xylazine (15 mg/kg). The inoculum was prepared in PBS and a maximum volume of 30 µL was administrated per mouse. Non-infected mice (control) received PBS only. At 72 h post-infections (p.i) and on the 14th day p.i, mice were euthanized and the bronchoalveolar lavage (BAL) and lungs were collected. Blood was collected from tail vein on days 0, 7, 15, and 30 post-infection for antibody quantification. Mice were also weighed 24, 48, and 72 h p.i. and on days 7 and 14 post-infection. Mice that reached the humane endpoints and showed signs of suffering (determined by 20% body weight loss, slow movements, alteration of general appearance, and changes in posture and behavior) were humanely euthanized by overdose of ketamine (80 mg/kg) and xylazine (15 mg/kg) followed by cervical dislocation and their deaths were recorded on the same day.

### 2.7. Enzyme Linked Immunosorbent Assay (ELISA) for IgM and IgG 

Blood collected from mice tail vein was centrifuged at 300× *g* for 5 min at 4 °C and the serum was used for quantifying the production of IgM and IgG antibodies by ELISA. ELISA plates were coated with EVs (5.0 µg/mL) or *P. brasiliensis* yeast cells (1.0 × 10^6^ cells/well) and, after incubation and washing, were blocked with 1% BSA in PBS. After washing, plates were incubated with mice sera diluted 1:100 in PBS. Plates were washed and then incubated with anti-mouse IgM-HRP or anti-mouse IgG-HRP (Southern Biotech, Birmingham, AL, USA). After washing, the reaction was developed by adding o-phenylenediamine dihydrochloride (OPD) (400 µg/mL—Sigma-Aldrich, Darmstadt, HE, Germany) and hydrogen peroxide in citrate buffer (pH = 5). The 96 well-flat bottom plates were read on a spectrophotometer at 490 nm wavelength. 

### 2.8. Fungal Burden Quantification

Lungs were disrupted in 1 mL of sterile PBS for the determination of the fungal burden. The bronchoalveolar lavage (BAL) was obtained by inserting a cannula on a small incision on the trachea of animals and washing the bronchoalveolar space with 2–3 mL of PBS. Both BAL and lung homogenate were plated on Brain Heart Infusion agar (BHI) (DIFCO, Lawrence, KS, USA) supplemented with 4% (*v*/*v*) heat-inactivated FBS, 5% *P. brasiliensis* B339 culture supernatant and 40 mg/L gentamicin (Sigma-Aldrich, St. Louis, MO, USA) [17]. Plates were incubated at 37 °C and colonies were counted after 7–15 days for determination of the colony-forming units (CFU) per gram of tissue or mL of BAL.

### 2.9. Myeloperoxidase (MPO) and N-Acetylglucosaminidase (NAG) Activity Evaluation

MPO and NAG activities were evaluated in the lung parenchyma at both timepoints, 72 h and on day 14 p.i. For MPO activity, lung tissue was homogenized in pH 4.7 buffer (0.1 M NaCl, 0.02 M Na_3_PO_4_, and 0.015 M Na_2_-EDTA), centrifuged, and the remaining pellet incubated with a 0.2% NaCl solution followed by incubation with 1.6% NaCl solution supplemented 5% of glucose. The solution was then centrifuged and the pellet resuspended in buffer containing 0.05 M Na_3_PO_4_ and 0.5% hexadecyltrimethylammonium bromide. The suspension was submitted to three freeze-thaw steps using liquid nitrogen, centrifuged, and the supernatant incubated with 1.6 M tetramethylbenzidine containing 0.002% H_2_O_2_ to evaluate the MPO activity. The reaction was stopped by the addition of 1 M H_2_SO_4_ and the 96-well flat-bottom plates were read on a spectrophotometer at 450 nm wavelength [18]. The results were expressed as relative units of MPO.

For NAG activity assay, lung parenchyma was homogenized in the same buffers as previously described. However, after incubation with the solution of 1.6% NaCl supplemented with 5% glucose and centrifugation, the pellet obtained was resuspended in a solution of 0.9% NaCl containing 0.1% (*v*/*v*) of Triton X-100 at 4 °C. Then, the suspension was used to measure the NAG activity using a 96-well-flat bottom plate. The supernatant was incubated with p-nitrophenyl-N-acetyl-D-glucosamine (Sigma-Aldrich, St. Louis, MO, USA) and dissolved in citrate/phosphate buffer (0.1 M citric acid and 0.1 M Na_2_PO_4_, pH 4.5) at a final concentration of 2.24 mM. The reaction was stopped by the addition of 0.2 M glycine buffer (pH 10.6) and quantified at 405 nm wavelength in a spectrophotometer [19]. The results were expressed as relative units of NAG.

### 2.10. Cytokine Analysis

ELISA using lung homogenates and supernatant from splenocyte cultures was performed for the measurement of cytokine production. Lung parenchyma was homogenized in PBS containing protease inhibitor cocktail (0.1 mM phenylmethylsulfonyl fluoride, 0.1 mM benzethonium chloride, 10 mM ethylenediaminetetraacetic acid (EDTA), and 20 KI aprotinin A) and 0.05% Tween. The level of the cytokines TNF, IFN-γ, and IL-17 were determined in the supernatant using commercially available antibodies according to manufacturer’s instructions (R&D Systems, Minneapolis, MN, USA). The results were expressed as pg/100 mg of tissue. 

### 2.11. Histopathological Analysis 

Lung tissue was fixed in 10% formalin and embedded in paraffin blocks. Then, for histopathological evaluation, tissue sections (5 mm thick) were stained with Hematoxylin and Eosin (H and E). The histopathological score was evaluated using a light microscope and sections were captured with a digital camera (DEI-470; Optronics, Goleta, CA, USA) connected to a microscope (IX70; Olympus, Center Valley, PA, USA. The assessed parameters were inflammatory infiltrate, distribution of inflammation, interstitial/alveolar edema, necrosis, and hemorrhage [20].

### 2.12. Flow Cytometry Analysis 

Flow cytometer analysis of the cells evoked by immunization with EVs from *P. brasiliensis* was performed on the 7th day p.i. Bronchoalveolar cells were harvested and labeled with a panel of monoclonal antibodies (anti-CD4/FITC, anti-CD8a/PE, anti-CD3/PE-Cy5, anti-CD44/Alexa 647, and anti-NK1.1/PE-Cy7) (all from Biolegend Inc., San Diego, CA, USA). Data were acquired on a FACSCanto II (Becton Dickinson, Franklin Lakes, NJ, USA) and analyzed using FlowJo v10 software (TreeStar Inc., Ashland, OR, USA. Limits for the quadrant markers were always set based on the negative population stained with isotype controls. Results were presented as cell number per mL of BAL (×10^5^).

### 2.13. Statistical Analysis

Statistical analyses were performed using the GraphPad Prism software (GraphPad Software, La Jolla, CA, USA), using a One-Way analysis of variance (ANOVA) and Newman–Keuls multiple comparison tests. The histopathological score was analyzed by Kruskal–Wallis, a nonparametric test. A *p* value <0.05 was considered to be significant, and the results are shown as means ± standard deviation.

## 3. Results

### 3.1. Immunization with P. brasiliensis-Derived EVs Induces Antibody Production In Vivo and Cytokine Release upon Ex Vivo Restimulation 

**Figure 1:**
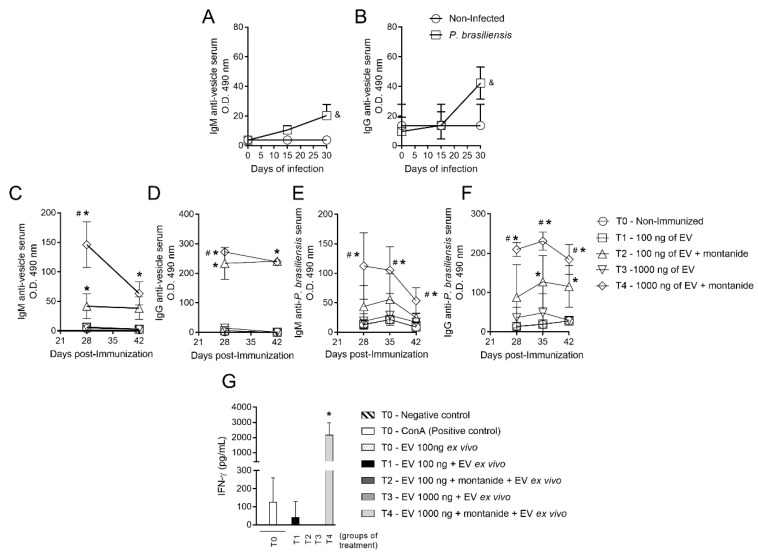
Standardization of the immunization with extracellular vesicles. Mice infected with *P. brasiliensis* yeast cells without immunization by EVs produce antibodies that recognize proteins carried by EVs from *P. brasiliensis*. IgM against EV-derived protein (**A**). IgG against EV-derived protein (**B**). ^&^ *p* ≤ 0.05, statistically significant compared to non-infected. Evaluation of IgM and IgG levels on serum after immunization with EVs in mice not infected with *P. brasiliensis*. IgM anti-EV-derived protein (**C**). IgG anti-EV-derived protein (**D**). IgM anti-*P. brasiliensis* (**E**). IgG anti-*P. brasiliensis* (**F**). Group T0 refers to non-immunized mice (control). Groups T1, T2, T3, and T4 refer to mice immunized with the indicated amount of EV-derived protein in the presence or absence of adjuvant. * *p* ≤ 0.05, statistically significant compared to non-immunized (T0), T1, and T3 groups. ^#^ *p* ≤ 0.05, statistically significant compared to the T2 group. Splenocytes from mice immunized with EVs but not infected with *P. brasiliensis* were incubated ex vivo with EVs to evaluate of IFN-γ production. (**G**). * *p* ≤ 0.05, statistically significant compared to non-immunized (T0), T1, T2, and T3 groups. Data represents one experiment. *n* = 6 animals per group.

In order to demonstrate that EVs contain antigens relevant for an antibody-mediated immune response to *P. brasiliensis*, mice were immunized with different concentrations of EV protein. Subsequently, ELISA was performed to assess the presence of antibodies against EVs from *P. brasiliensis* protein in mice serum. Production of IgM and IgG antibodies targeting protein antigens present in EVs increased both on the 15th and 30th day post-infection, reaching its highest levels on the 30th day post-infection (Figure 1A,B).

As a means to determine the optimal dose of EV-derived protein for mouse immunization, distinct protein concentrations were injected in different mouse groups either with or without an adjuvant. The concentrations of 100 ng and 1000 ng of EV protein in the presence of Montanide (adjuvant) led to an increase in IgM and IgG production against both EV-derived protein and *P. brasiliensis* cell-derived protein on the timepoints tested (Figure 1C–F). This difference was not observed in the absence of the adjuvant (Figure 1C–F). Antibody production was highest in mice immunized with the 1000 ng concentration with Montanide (Figure 1C–F). Additionally, whilst IgM levels against *P. brasiliensis* cell protein decreased after the 28th day post-immunization, levels of IgG directed to *P. brasiliensis* cell protein remained detectable until the 42nd day (Figure 1E,F). However, neither the 100 ng nor the 1000 ng dose of EV protein, in the absence of Montanide, induced an increase in the production of these antibodies (Figure 1C–F). IgG production was significant in the presence of adjuvant against both EV protein and *P. brasiliensis* yeast cells though the highest signal observed for IgG was against EV protein (Figure 1D,F).

Production of IFN-γ by splenocytes ex vivo was evaluated after the immunization protocol standardization. Following exposure to 100 ng of EV-derived protein, only splenocytes obtained from mice immunized with 1000 ng of EV-derived protein and adjuvant produced detectable amounts of IFN-γ (Figure 1G). 

### 3.2. Immunization with P. brasiliensis-Derived EVs Ameliorates Histopathological Parameters and Reduces Fungal Burden in Lung Tissue 72 h Post-Infection by P. brasiliensis

**Figure 2:**
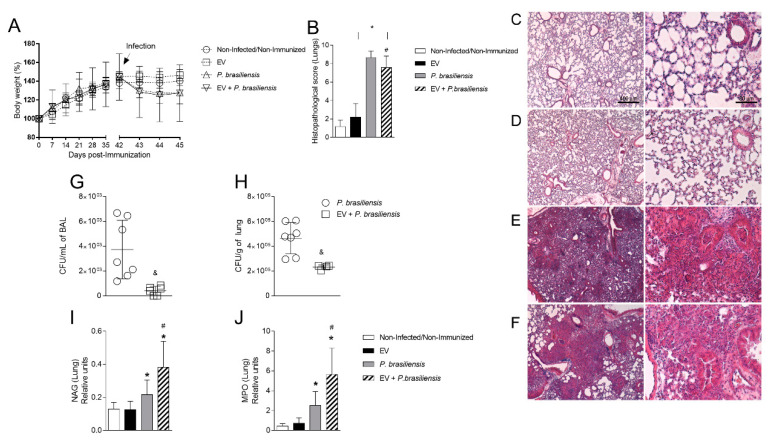
Immunization with EVs from *P. brasiliensis* alters tissue architecture and reduces fungal burden in the lung tissue and the inflammatory profile of lung tissue 72 h post-infection. Body weight (**A**). Histopathological score (**B**). Lung histology ((**C**–**F**), scale bars—left 400 µm and right 20 µm)—non-infected/non-immunized group (**C**), EV group (**D**), *P. brasiliensis* group (**E**), EV + *P. brasiliensis* group (**F**). Fungal burden in BAL (**G**). Fungal burden in lung tissue (**H**). NAG (**I**). MPO (**J**). ^&^ *p* ≤ 0.05 compared to *P. brasiliensis* group. * *p* ≤ 0.05 compared to non-infected/non-immunized and EV groups. ^#^ *p* ≤ 0.05 compared to *P. brasiliensis* group. Non-infected/non-immunized (control). Immunization for 42 days with two doses of 1000 ng of EV protein with adjuvant (boost dose, without adjuvant, given 14 days after the first dose). Data represents the mean of two independent experiments. *n* = 8 animals per group.

To investigate the participation of *P. brasiliensis*-derived EVs in the protection of pulmonary lesions caused by the fungal infection, mice were immunized with 1000 ng of EV protein with adjuvant, with an immunization boost 14 days after the first dose, followed by infection with *P. brasiliensis* yeast cells. Bodyweight, lung histopathological profile, fungal burden, and accumulation of macrophages and neutrophils were evaluated 72 h post-infection by *P. brasiliensis*. There was no weight loss after immunization (Figure 2A). Body weight reduction was observed only after infection by *P. brasiliensis* yeast cells (Figure 2A). Histopathological analysis (Figure 2B–F) showed that *P. brasiliensis* yeast cells altered lung architecture, given that it induced intense leukocyte infiltration, alveolar edema, and tissue necrosis after infection (Figure 2E). Immunization with EVs (EV + *P. brasiliensis* group), however, partially reduced the histopathological lesion as compared to *P. brasiliensis* group (Figure 2B). Additionally, the immunized group had reduced fungal burdens both in BAL and in lung tissue (Figure 2G,H) compared to the infection-only group. Increased levels of MPO and NAG enzymes activity (quantitative index for the measurement of neutrophils and macrophages recruitment to tissue, respectively) in the lung tissue after the infection was observed in the groups *P. brasiliensis* and EV + *P. brasiliensis* as compared to control and EVs groups, 72 h post-infection (Figure 2I,J). However, the highest levels were observed in the EV + *P. brasiliensis* group (Figure 2I,J).

### 3.3. Immunization with P. brasiliensis-Derived EVs Ameliorates Histopathological Parameters and Reduces Fungal Burden in Lung Tissue 14 Days Post-Infection with P. brasiliensis Yeast Cells

**Figure 3:**
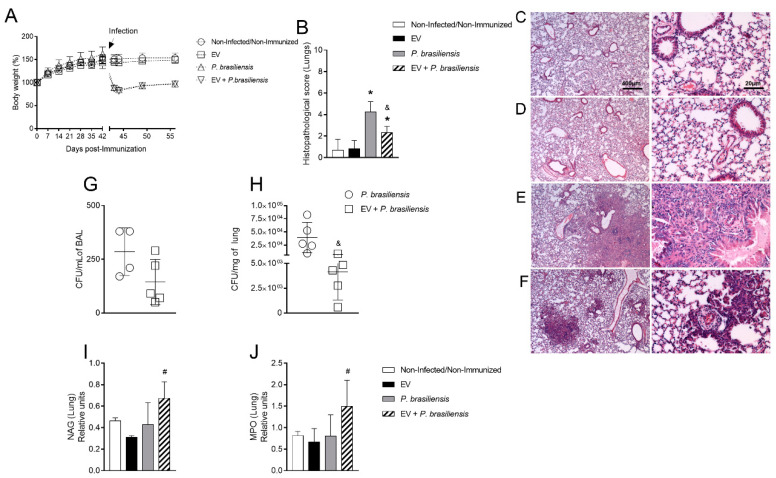
Immunization with EVs from *P. brasiliensis* alters tissue architecture and reduces fungal burden at lung tissue and the inflammatory profile of lung tissue at day 14 post-infection. Body weight (**A**). Histopathological score (**B**). Lung histology (**C**–**F**, scale bars—left 400 µm and right 20 µm). Non-infected/non-immunized group (**C**), EV group (**D**), *P. brasiliensis* group (**E**), EV + *P. brasiliensis* group (**F**). Fungal burden in BAL (**G**). Fungal burden at lung tissue (**H**). NAG assay (**I**). MPO assay (**J**). ^&^ *p* ≤ 0.05 compared to *P. brasiliensis* group. ^#^ *p* ≤ 0.05 compared to non-infected/non-immunized, EV, and *P. brasiliensis* groups. * *p* ≤ 0.05 compared to the non-infected/non-immunized and EV groups. Non-infected/non-immunized (control). Immunization for 42 days with two doses of 1000 ng of EV protein with adjuvant (boost dose, without adjuvant, given 14 days after the first dose). Data represents one experiment. *n* = 6 animals per group.

In order to investigate whether *P. brasiliensis*-derived EVs ameliorate pulmonary tissue lesions 14 days post-infection, mice were immunized with 1000 ng of EV protein with adjuvant, with a boost given 14 days after the first dose, followed by infection with *P. brasiliensis* yeast cells. Body weight, histopathological analysis and fungal burden were evaluated at 14 days after infection with *P. brasiliensis*. Mice presented no bodyweight changes after immunization (Figure 3A). Infection with *P. brasiliensis* reduced bodyweight in the *P. brasiliensis* and EV + *P. brasiliensis* groups. Infection by *P. brasiliensis* yeast cells was associated with diffuse mononuclear cell infiltrate, alveolar edema, tissue necrosis and hemorrhage, altered lung architecture, and increased histopathological score compared to control and EV groups (Figure 3B–F). Among infected groups, reduced histopathological scores were observed in the EV + *P. brasiliensis* group compared to *P. brasiliensis* group (Figure 3B). Considering the fungal burden, whilst there was no significant difference in the BAL, a significant reduction was observed in the lung tissue between the groups (Figure 3G,H). There was no difference in the levels of NAG and MPO enzymes among the control, EV, and *P. brasiliensis* groups. Interestingly, the highest levels of both enzymes were observed in the group EV + *P. brasiliensis* (Figure 3I,J). 

### 3.4. Immunization with P. brasiliensis-Derived EVs Induces Mobilization of Activated T lymphocytes and NKT Cells upon Challenge

**Figure 4:**
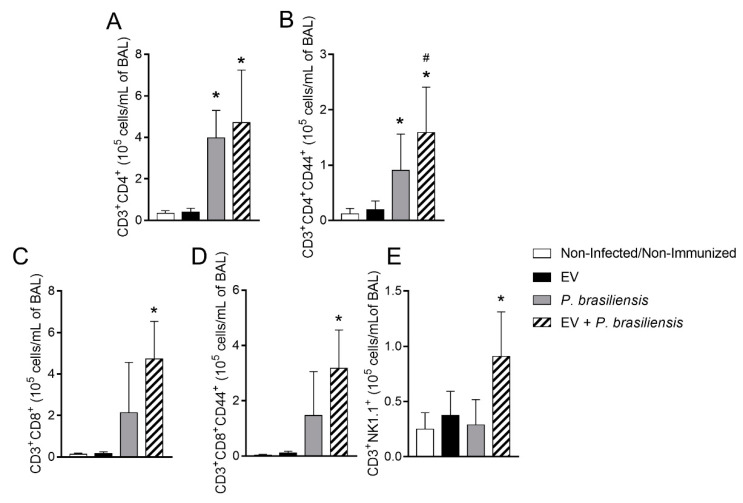
Memory CD4^+^ and CD8^+^ T-cells and NKT cells recalled by *P. brasiliensis* infection at day 7 post-infection. CD3^+^ CD4^+^ T lymphocytes (**A**). CD3^+^ CD4^+^ CD44^+^ T lymphocytes (**B**). CD3^+^ CD8^+^ T lymphocytes (**C**). CD3^+^ CD8^+^ CD44^+^ T lymphocytes (**D**). CD3^+^ NK 1.1^+^ cells (**E**). * *p* ≤ 0.05 compared to non-infected/non-immunized and EV groups. ^#^ *p* ≤ 0.05 compared to *P. brasiliensis* group. Non-infected/non-immunized (control). Immunization for 42 days with two doses of 1000 ng of EV protein with adjuvant (boost dose, without adjuvant, given 14 days after the first dose). Mice were infected on day 42 post-immunization. BAL was collected 7 days post-infection with *P. brasiliensis* for flow cytometry analysis. *n* = 6 animals per group. Data represents one experiment.

Flow cytometry analysis was performed on the 7th day post-infection in order to evaluate the cell influx to the site of infection and how this was influenced by immunization with EVs from *P. brasiliensis*. We observed BAL cells expressing the activation marker CD44 and surface markers specific for T lymphocytes (CD4 and CD8) on day 7 post-infection (Figure 4A–E, Figure S1). Mice previously immunized and infected (EV + *P. brasiliensis*) displayed increased counts of activated CD4-positive T cells in the BAL upon infection (Figure 4B). There was also an increase in numbers of CD8^+^ memory T cells and NKT cells after immunization with EVs and subsequent infection with *P. brasiliensis* (Figure 4D,E). 

### 3.5. Mice Immunized with EVs from P. brasiliensis Had Higher Production of Antibodies Specific to Both EV-Derived Protein and P. brasiliensis Cells

**Figure 5:**
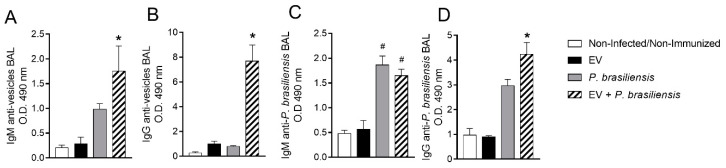
Immunization with EVs from *P. brasiliensis* induces augmentation of IgG against extracellular vesicles and *P. brasiliensis* yeast cell protein on day 7 post-infection. IgM anti-EV-derived protein (**A**). IgG anti-EV-derived protein (**B**). IgM anti-*P. brasiliensis* (**C**). IgG anti-*P. brasiliensis* (**D**). * *p* ≤ 0.05 compared to non-infected/non-immunized, EV and *P. brasiliensis* groups. ^#^ *p* ≤ 0.05 compared to non-infected/non-immunized and EV groups. Non-infected/non-immunized (control). Immunization for 42 days with two doses of 1000 ng of EV protein with adjuvant (boost dose, without adjuvant, given 14 days after the first dose). *n* = 6 animals per group. Data represents one experiment.

ELISAs were performed to investigate the production of IgM and IgG in the BAL after immunization with EVs from *P. brasiliensis* (Figure 5A–D) that would be specific against EV-derived protein and *P. brasiliensis* cells. Control and EVs groups presented low levels of IgM or IgG to EV-derived protein and to *P. brasiliensis* yeast cells (Figure 5A–D). These low levels of IgM/IgG did not reflect the results observed in Figure 1 in the EV group, possibly due to the sample type (serum × BAL) and timepoint of analysis (days 28, 35 and 42 post first dose of immunization in Figure 1 × day 49 post first dose of immunization in Figure 5). The group EV + *P. brasiliensis* displayed the highest production of IgM and IgG to EV-derived protein (Figure 5A,B). Both *P. brasiliensis* and EV + *P. brasiliensis* groups presented an increase in the production of IgM and IgG to *P. brasiliensis* protein when compared to the control and EV groups (Figure 5C,D). However, the highest levels of anti-*P. brasiliensis* IgG were observed in the EV + *P. brasiliensis* group (Figure 5D).

### 3.6. Mice Immunized with EVs from P. brasiliensis Had Higher Levels of Cytokines Important to Control P. brasiliensis Infection 

**Figure 6:**
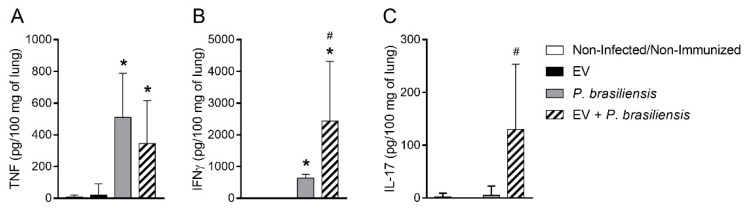
Immunization with EVs from *P. brasiliensis* increases cytokine levels 72 h post-infection. TNF (**A**). IFN-γ (**B**) and IL-17 (**C**). * *p* ≤ 0.05 compared to non-infected/non-immunized and EV groups. ^#^ *p* ≤ 0.05 compared to non-infected/non-immunized, EV and *P. brasiliensis* groups. Non-infected/non-immunized (control). Immunization for 42 days with two doses of 1000 ng of EV protein with adjuvant (boost dose, without adjuvant, given 14 days after the first dose). Data represents the mean of two independent experiments. *n* = 8 animals per group.

ELISA was performed to investigate the cytokine levels in lung homogenate after immunization with EVs from *P. brasiliensis* (Figure 6A–C). TNF, IFN-γ and IL-17 levels were either below detection limits or low in control and immunized mice (EV) groups (Figure 6A–C). Lung TNF production was increased in *P. brasiliensis* and EV + *P. brasiliensis* groups (Figure 6A). In a similar manner, pulmonary IFN-γ levels were increased in *P. brasiliensis* and EV + *P. brasiliensis* groups (Figure 6B). However, the EV + *P. brasiliensis* group presented higher IFN-γ levels (Figure 6B). In addition, IL-17 detection was observed only in the EV + *P. brasiliensis* group (Figure 6C). 

## 4. Discussion

The present study demonstrates that immunization with EVs from *P. brasiliensis* induces a protective effect that is associated with reduction in lung fungal burden in a PCM murine model. Although the in vitro production of EVs by *P. brasiliensis* has been previously demonstrated, there is still little information about its in vivo relevance [13]. In the present work, we demonstrated the generation of antibodies against products carried by EVs from the fungal cells. The induction of IgM and IgG to EV-derived protein suggests a specific antibody response against the fungus and its products transported within EVs.

Montanide ISA 50 is an oil-based adjuvant and was chosen in this study due to its depot effect after emulsion with the immunogen. This adjuvant with the antigen also drives the activation of specific T cells involved with the production of antibodies [21,22]. Thus, the optimization of immunization with EVs indicates the need of a booster 14 days after the first dose of EVs. The dose of 1000 ng of EV-derived protein with Montanide was the optimal combination, inducing the highest levels of serum IgM and IgG antibodies observed against *P. brasiliensis*. These results show that the immunization protocol presented here efficiently triggers the production of specific antibodies targeting both proteins located within EVs and on the fungal surface. In addition, this dose was able to generate IFN-γ-releasing memory T cells, as demonstrated by recall assays involving splenocyte exposure to EV antigens. Thus, EVs induced antigen-specific cells, which were fully functional, by producing effector cytokines during proliferation. It is worth mentioning that IFN-γ has a role in stimulating T cell proliferation and in the generation of effector and memory T cells [23]. IFN-γ is also involved in macrophage activation and in fungal replication control during PCM [24]. Interestingly, IFN-γ and TNF were also found in the culture supernatant of splenocytes from mice immunized with the DNA vaccine pcDNA3-P10 [5]. Thus, the pattern of cytokines released by splenocytes from mice immunized with EVs plus Montanide is consistent with a Th1-biased T-cell immune response which is predictive of a good clinical response.

The results presented in this study indicate that the protocol of immunization with *P. brasiliensis* EVs enhanced the resolution of the pulmonary infection, presenting reduced tissue injury. In addition, animals immunized with EVs in Montanide showed a significant reduction in lung fungal burden compared to the control group, both 72 h and 14 days post-infection by *P. brasiliensis*. Similarly, the studies of de Munoz et al. [4], Amorin et al. [5], and Holanda et al. [7], also demonstrated improvement in tissue architecture maintenance and fungal load reduction. Additionally, NAG and MPO assay results showed accumulation of inflammatory cells in the lungs, which are important cells to control the fungal burden, preventing tissue architecture loss secondary to infection [25,26].

Our data suggest that immunization with EVs efficiently induces mobilization of protective EV-specific CD4^+^ memory T lymphocytes. These results were similar to those reported by Holanda et al. [7] using VLP carrying multiple copies of P10 peptide as a vaccine. Moreover, immunization with EVs also seemed to induce protective EV-specific CD8^+^ memory T lymphocytes mobilization, and the participation of these cells in the reduction in the fungal load during pulmonary PCM has been previously discussed [26]. For instance, Calish et al. [26] reported the importance of CD8α^+^ T cells in fungal containment during pulmonary PCM. It was also demonstrated that these cells produce greater amounts of IL-10 compared to CD4^+^ T cells during *P. brasiliensis* infection, suggesting a different role for CD8^+^ T cells, which presented a T-helper profile rather than a cytotoxic one after stimulation with *P. brasiliensis* [27,28]. Further, the present work shows that immunization with EV induces an increase in NKT cell number in lungs after infection, a result also reported by Munoz et al. [4]. NKT cells are a T lymphocyte specialized subtype that recognize lipid-based antigens present in bacterial and fungal cells, such as *Bacteroides fragilis* and *Aspergillus fumigatus*, respectively [29,30]. Therefore, the increment of NKT cell counts in immunized mice indicates that these cells compose an important immune mechanism in host defense during PCM as well [4].

Immunized mice exhibited high levels of both IgM and IgG antibodies targeting EV-derived protein and *P. brasiliensis* cells, strengthening the importance of the production of specific antibodies against *P. brasiliensis* protein to control the fungal infection. Interestingly, even though antibody response does not play an essential role in the containment of the *P. brasiliensis* infection, some reports have pointed to IgG2 subclass participation in infection resolution [15,31]. Additionally, immunization with EVs also induced the production of the pro-inflammatory cytokines TNF, IFN-γ, and IL-17, which have well-established roles in fungal replication control during PCM [6]. Further, these cytokines belong to a plethora of inflammatory mediators produced during the Th1 and Th17-biased T cell-immune responses, both pivotal to control fungal infections [26].

The cytokine panel in the present work includes those secreted by activated T-cells during antifungal response. Activation of CD4, CD8 T lymphocytes, and NKT cells indeed induced a broad spectrum of cytokines including IFN-γ and IL-17, which have a powerful effect on macrophage and neutrophil activation, which are important to containing fungal replication [25]. The importance of CD4^+^ T lymphocytes producing IFN-γ and IL-17 to control fungal infection was observed, for instance, in models of infection by *Pneumocystis*, *H. capsulatum*, *Cryptococcus neoformans*, *P. brasiliensis*, *Candida albicans*, and *A. fumigatus* [7,31,32,33,34]. CD8^+^ T-cells, on the other hand, act to control fungal infection either by cell-mediated cytotoxic activity or by producing IFN-γ and TNF. Their participation is necessary to clear infected macrophages with *H. capsulatum* yeast cells and to promote full protection against coccidioidomycosis. Moreover, CD8^+^ T lymphocytes producing IL-17 provide protection to infection by *Blastomyces dermatitidis* and *H. capsulatum*, via neutrophil activation [31,33]. EV-immunized mice presented increased macrophage and neutrophil mobilization to lung parenchyma upon infection. Together, these findings suggest that immunization with *P. brasiliensis*-derived EV is able to induce protective effector responses that protect from PCM development upon infection.

Our findings suggest a new role for EVs from *P. brasiliensis* as modulators of the host immune system, and provide insights into their use as immunizing agents. Compared to peptide-based immunization or other immunization methods, EVs likely play a role in cross-priming, acting, therefore, as a natural mechanism for transporting antigens in the host. However, some conceptual and practical issues need to be addressed prior to their potential application in clinical trials. Nevertheless, the present data indicates that EVs may provide an effective source of antigen for immunization, which is very important for *P. brasiliensis* infection considering its relevance in Latin American countries, mainly Brazil, where this mycosis is endemic. Although for this mycosis antifungal drugs have been used with good clinical response, the duration of the treatment is a clinical burden. In addition, prophylactic and immune-therapeutic induction of protective immune responses is a highly costly approach, justifying the importance of the research of new therapeutic methods such as EVs.

In conclusion, immunization with EVs in the presence of adjuvant provided effective control of fungal burden in the lungs of *P. brasiliensis*-infected mice. This method also improved the induction and mobilization of activated T lymphocytes and NKT cells, the production of proinflammatory cytokines, mobilization of effector cells and the protection from histologic damage. Thus, the results point to EVs as being an important, cost-effective, and novel tool for the development of vaccines and the prevention of PCM.

## Data Availability

The data underlying this study will be shared on request to the corresponding author.

## Supplementary Materials

The following are available online at https://www.mdpi.com/article/10.3390/cells10071813/s1, Figure S1: Gating strategy for FACS analysis of bronchoalveolar lavage fluid (BALF).
