# Supplementary material for: Protective Response in Experimental Paracoccidioidomycosis Elicited by Extracellular Vesicles Containing Antigens of Paracoccidioides brasiliensis
Source: Cells. 2021 Jul 17;10(7):1813. doi: 10.3390/cells10071813 (PMC8304155; doi:10.3390/cells10071813)
Supplement: Supplementary file 1 [file cells-10-01813-s001.zip › cells-1278093-supplementary.pdf]

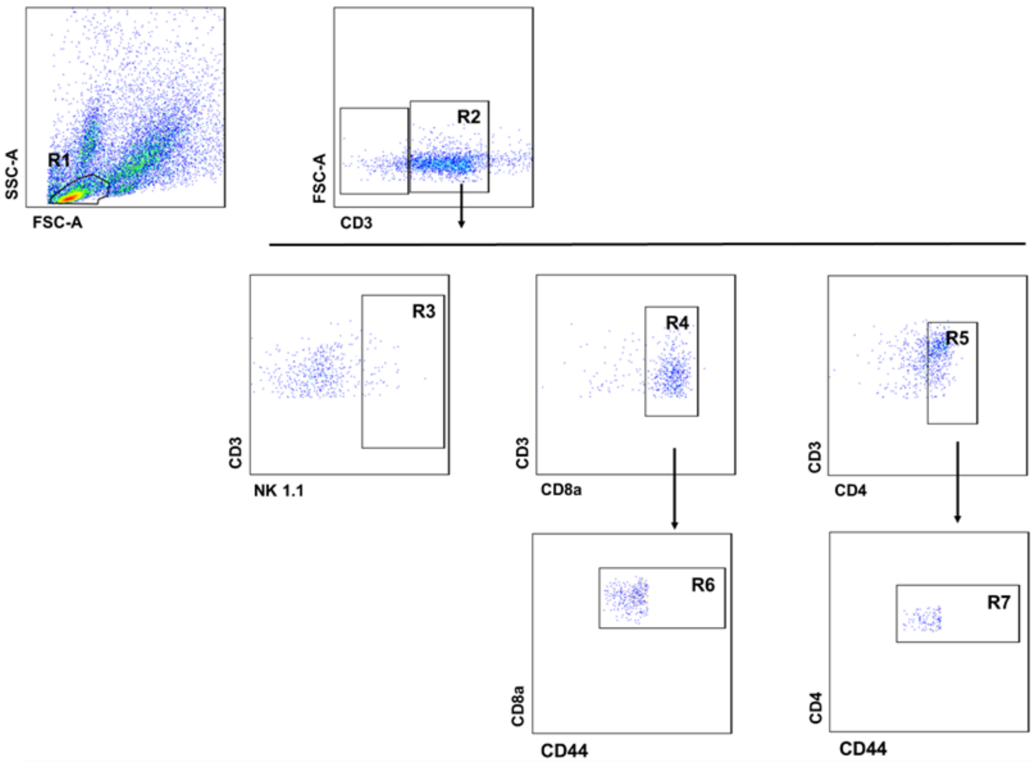

Figure S1. Gating strategy for FACS analysis of BALF. Cells (R1) were stratified according to size and granularity (R2) and then according to CD3 expression. CD3$^+$ NK 1.1$^+$ cells (R3). CD3+ CD8$^+$ T lymphocytes (R4). CD3$^+$ CD8$^+$ CD44$^+$ T lymphocytes (R6). CD3$^+$ CD4$^+$ T lymphocytes (R5). CD3$^+$ CD4$^+$ CD44$^+$ T lymphocytes (R7).
